# Absorption and Interaction of the Main Constituents from the Traditional Chinese Drug Pair Shaoyao-Gancao via a Caco-2 Cell Monolayer Model

**DOI:** 10.3390/molecules171214908

**Published:** 2012-12-13

**Authors:** Yan Chen, Jinyan Wang, Lu Wang, Lianghui Chen, Qingqing Wu

**Affiliations:** Jiangsu Provincial Academy of Chinese Medicine, 100 Shizi Road, Nanjing 210028, Jiangsu, China; E-Mails: wwind924@yahoo.com.cn (J.W.); youleiwuheng@yahoo.com.cn (L.W.); clhatm@163.com (L.C.); wqq18314@126.com (Q.W.)

**Keywords:** paeoniflorin, liquiritin, glycyrrhizic acid, extract, absorption, interaction, Caco-2 cell monolayer model

## Abstract

Shaoyao-Gancao (*Paeoniae Radix Alba* and *Glycyrrhizae Radix et Rhizoma*) is a traditional Chinese drug pair widely used in decoctions for relieving pains, especially abdominal pain. We aimed to determine the intestinal absorption and interaction of three active compounds (glycyrrhizic acid, liquiritin, and paeoniflorin) in this drug pair. We investigated the transport of these compounds across intestinal epithelial cells by using the Caco-2 cell monolayer in both the apical-to-basolateral (A-B) and B-A directions. All compounds could only travel through the Caco-2 cell monolayer at a low level when the cells were treated with single component solutions. In the presence of verapamil, an inhibitor of P-glycoprotein (P-gp), the absorptive permeability (P_AB_) of paeoniflorin and liquiritin increased significantly (*p* < 0.05) and efflux ratios decreased, while the absorption of glycyrrhizic acid did not change significantly, which indicated that paeoniflorin and liquiritin might be P-gp substrates. In addition, when liquiritin and glycyrrhizic acid in Gancao extract and paeoniflorin in Shaoyao extract were examined, P_AB_ of paeoniflorin and liquiritin were significantly higher, while glycyrrhizic acid retained the same absorption level compared to the corresponding single component solutions, which suggested that some certain ingredients in the extracts can promote the absorption of paeoniflorin and liquiritin, but not that of glycyrrhizic acid. Furthermore, compared to the results of treatment with individual extracts, treatment of cells with a mixture of the two extracts considerably increased (*p* < 0.05) the absorption of glycyrrhizic acid and paeoniflorin and showed no change in the absorption of liquiritin, which implied that the transport of glycyrrhizic acid and paeoniflorin is increased by some ingredients from the complementary drug in the drug pair, while that of liquiritin remains unaffected.

## 1. Introduction

Traditional Chinese medicines (TCMs) are natural therapeutic remedies that have been widely used for thousands of years [1]. The use of TCMs involves two important features: formulated prescription and oral administration [2]. Most TCMs are prescribed using multi-ingredient formulas, and it is widely accepted that multiple constituents are responsible for their particular bioactivities [3]. Meanwhile, it is well known that oral administration is the main route for applying TCMs and they should be absorbed in the gastrointestinal tract to exhibit pharmacologic effects [4]. Therefore, to understand the principles governing the prescription of TCMs, the interactions of the constituents of TCMs during the intestinal absorption process should be primarily studied. Because drug pairs are the simplest form of TCM formulations, the study of drug pairs would be a substantial approach to comprehend the mechanisms underlying the formula of TCMs [5].

The Shaoyao-Gancao drug pair, a classical analgesic prescription, which consists of two herbs, Shaoyao (*Paeoniae Radix Alba*) and Gancao (*Glycyrrhizae Radix et Rhizoma*), is originated from Si-Ni-San derived from the Treatise on Febrile Diseases of Zhang Zhongjing and is widely used in decoctions for treating various inflammatory diseases, including gastritis, colitis, and hepatitis [6]. The characteristic active component in Shaoyao is paeoniflorin, a monoterpene glycoside with anti-inflammatory, antirheumatic, anticancer, and immunomodulatory properties [7,8,9]. Gancao has two main bioactive components, glycyrrhizic acid and liquiritin. Glycyrrhizic acid has various anti-inflammatory, antiallergic, anticarcinogenic, and immunomodulatory actions [10,11], while liquiritin is an active flavonoid glycoside often used to treat injuries or swelling because of its life-enhancing and detoxifying properties [12]. Additionally, paeoniflorin, glycyrrhizic acid, and liquiritin are used as markers to control the quality of Shaoyao and Gancao, respectively, in the Chinese Pharmacopoeia. 

Studies on the pharmacokinetic parameters (area under the curve [AUC] and elimination half-life [t_1/2_]) of the Shaoyao-Gancao drug pair reported to date indicate significant differences in the levels of the main compounds like paeoniflorin and glycyrrhizic acid in the plasma samples after oral administration of a single or mixed decoction of Shaoyao and Gancao [13]. However, few studies have used the Caco-2 cell model to clarify the intestinal absorption and mechanism of the compatibility of these active ingredients from the Shaoyao-Gancao drug pair. Therefore, to illustrate the principle of compatibility between the constituents of the Shaoyao-Gancao drug pair in terms of intestinal absorption, the intestinal epithelial transports of these compounds and their interactions in single component solution, single extract and mixture of two extracts were observed. Here, we used the Caco-2 cell monolayer model because this model is recognized by the Food and Drug Administration (FDA) as a viable model of human intestinal absorption and is routinely used to investigate drug absorption and metabolism [14]. Our findings showed that concomitant oral administration of Shaoyao and Gancao extracts significantly improved the absorption of glycyrrhizic acid and paeoniflorin, which to some extent provides the scientific evidence to support the synergistic effects of the Shaoyao-Gancao drug pair.

## 2. Results and Discussion

### 2.1. Transport of Paeoniflorin, Liquiritin, and Glycyrrhizic Acid across the Caco-2 Cell Monolayers

The absorption of paeoniflorin, glycyrrhizic acid and liquiritin, as well as the influence of P-glycoprotein (P-gp) inhibitor on them were investigated by the Caco-2 cell model. The concentrations of paeoniflorin, glycyrrhizic acid, and liquiritin were set at 50, 20 and 50 μM, respectively, because the traditional ratio of compatibility between Shaoyao and Gancao was 1:1 and the concentration of paeoniflorin, glycyrrhizic acid, and liquiritin in the mixture of Shaoyao and Gancao extract was close to the above-mentioned drug concentrations. Transport of these compounds in both apical-to-basolateral (A-B) and B-A directions was observed, and the absorptive permeability (P_AB_) and secretary permeability (P_BA_) of paeoniflorin, glycyrrhizic acid, and liquiritin were calculated. The P_AB_ values of paeoniflorin, glycyrrhizic acid, and liquiritin were 0.61 × 10^−6^ cm/s, 0.36 × 10^−6^ cm/s, and 0.73 × 10^−6^ cm/s, respectively, which were very low (Table 1). 

**Figure 1:**
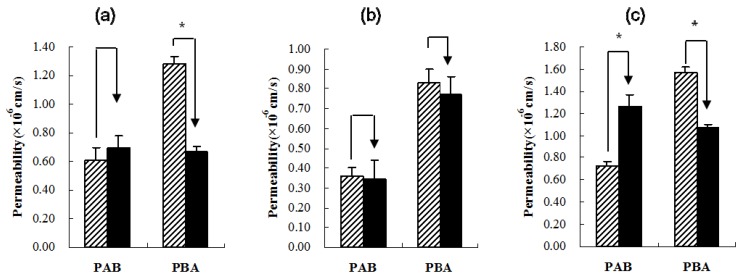
Effects of verapamil on permeability of paeoniflorin, glycyrrhizic acid, and liquiritin. (**a**): Paeoniflorin, (**b**): Glycyrrhizic acid, (**c**): Liquiritin. In the each column figure, slash column refers to absent of verapamil; black column refers to presence of verapamil. * *p* < 0.05, presence of verapamil *vs.* absence of verapamil.

The low P_AB_ values of these three compounds suggested that all of them had very poor absorption in the intestine. In contrast, the P_BA_ of the three compounds was significantly greater than their P_AB_ (*p* < 0.05). The efflux ratios (P_BA_/P_AB_) of paeoniflorin, glycyrrhizic acid and liquiritin were 2.10, 2.31 and 2.15, respectively, which were all >2, indicating that some transporters might be involved in the transport of these compounds [15]. Many reports have shown that P-gp is one of the main transporters that could influence drugs’ transport in the intestine [16]. To determine whether P-gp is involved in the transport of these compounds, their transports were studied in the presence of verapamil, a known inhibitor for P-gp [17]. After addition of verapamil (100 μM), the P_AB_ of liquiritin increased significantly (*p* < 0.05), while the P_BA_ of liquiritin and paeoniflorin decreased significantly (*p* < 0.05), which resulted in a decrease in the efflux ratio by 60.47% and 54.29% (Figure 1). This result indicated that paeoniflorin and liquiritin might be the substrates of P-gp, which was consistent with the findings reported by Liu [18,19]. On the other hand, the P_AB_ and P_BA_ of glycyrrhizic acid did not change significantly before and after the addition of verapamil, which indicated that P-gp might not be involved in the transport of glycyrrhizic acid. In addition to the P-gp transporter, multidrug resistance protein (MRP) and breast cancer resistance protein (BCRP) transporters are present in the intestinal tract of humans, which are also responsible for the transport and efflux of compounds or drugs [20]; however, further studies are required to clarify whether MRP or BCRP transporters were involved in the transport of glycyrrhizic acid. Moreover, no metabolite of these three compounds was found under the experimental conditions used in this study.

### 2.2. Transport of Paeoniflorin in the Shaoyao Extract and Glycyrrhizic Acid and Liquiritin in the Gancao Extract

Permeability of paeoniflorin in the Shaoyao extract and glycyrrhizic acid and liquiritin in the Gancao extract is shown in Table 2. At the same concentration of the compound, the P_AB_ values of paeoniflorin in the Shaoyao extract and liquiritin in the Gancao extract were higher than those of the single compound, while the P_BA_ values of both compounds showed no significant change and thus the efflux ratios were reduced (Table 2). 

This observation indicated that some ingredients in the Shaoyao or Gancao extract could inhibit the efflux effect of P-gp, which promoted the absorption of paeoniflorin and liquiritin. It seemed that the ingredients in Gancao extract could not influence the absorption of glycyrrhizic acid, as there was no big difference of P_AB_ and P_BA_ between the single component and extract.

### 2.3. Interaction of Paeoniflorin, Glycyrrhizic Acid, and Liquiritin in the Shaoyao-Gancao Extract

The concentrations of paeoniflorin, glycyrrhizic acid, and liquiritin in the mixture of Shaoyao-Gancao extract were 50, 22, and 54 μM, respectively, to maintain the concentration of these compounds the same as that in the Shaoyao or Gancao extract. The P_AB_ of paeoniflorin in the mixture was significantly higher than that in the Shaoyao extract, which indicated that ingredients in Gancao might promote the absorption of paeoniflorin (Table 3). Besides, compared to the P_AB_ of glycyrrhizic acid in the Gancao extract, the P_AB_ in the mixture increased dramatically (over two times) and the efflux ratio decreased by more than 1-fold, which indicated that the ingredients in Shaoyao extract might promote the absorption of glycyrrhizic acid. Moreover, because the P_AB_ and P_BA_ values of liquiritin in the mixture and in the Gancao extract alone were not significantly different, we thought that the ingredients in Shaoyao might have little influence on the absorption of liquiritin. Our findings clearly explained the reason underlying the significant increase in the plasma concentration of paeoniflorin and glycyrrhizic acid and high bioavailability after oral administration of a mixed decoction of Shaoyao and Gancao.

## 3. Experimental

### 3.1. Materials and Reagents

Paeoniflorin, glycyrrhizic acid and liquiritin (all purity >98%) were purchased from the National Institute for the Control of Pharmaceutical and Biological Products (Beijing, China). Hanks’ balanced salt solution (HBSS, powder form) and testosterone were obtained from Sigma-Aldrich (St. Louis, MO, USA). Fetal bovine serum was purchased from HyClone (Logan, UT, USA). Shaoyao (*Paeonia lactiflora* Pall.) and Gancao (*Glycyrrhiza uralensis* Fisch.) were supplied by Anhui Jingquan Chinese Herbal Medicine Company (Anhui, China) and were identified by Professor Dekang Wu, School of Pharmacy, Nanjing University of Chinese Medicine (Nanjing, China). All other materials (typically analytical grade or better) were used as received.

### 3.2. Cell Culture 

Caco-2 cells were donated from Dr Ming Hu (University of Houston, TX, USA). Cells were cultured in a humidified atmosphere of 5% CO_2_ and 95% air at 37 °C. The Dulbecco’s modified Eagle’s medium was supplemented with 10% (v/v) fetal bovine serum, 1% nonessential amino acids, 100 U/mL penicillin, and 100 µg/mL streptomycin. When the cell culture reached 80% confluence, it was rinsed with phosphate-buffered saline and split using trypsin. For transport experiments, the cells were seeded on 3 μm porous polycarbonate cell culture inserts from Nunc, which has a surface area of 4.2 cm^2^ at a density of 2,500,000 cells/cm^2^. The culture media was replaced every other day. The monolayers were ready for experiments from 19 to 22 days after seeding. Well-developed Caco-2 cell monolayers with transepithelial electrical resistance (TEER) values greater than 400 Ω × cm^2^ were used for the experiment.

### 3.3. Sample Preparation

Paeoniflorin, glycyrrhizic acid, and liquiritin were dissolved in dimethyl sulfoxide (DMSO)-ethanol (v/v = 1/1) to prepare the stock solution (10 mM) for each single compound. The above-mentioned solutions were further diluted with Hank’s balanced salt solution (HBSS, pH = 7.4) to obtain a series of working standard solutions, and the final concentrations of paeoniflorin, glycyrrhizic acid, or liquiritin in the transport samples were 50 µM, 20 µM, or 50 µM, respectively. Moreover, the final concentrations of the organic solvent of different samples were controlled below 0.5% to ensure the safety to the cells.

Dry Shaoyao (100 g) or Gancao (100 g) herb materials were extracted twice with boiling water for 1 h each time, and the solutions were filtered through gauze. Then, the filtrates were merged and concentrated to 100 mL as the Shaoyao extract (1 g/mL) containing paeoniflorin (22.41 mg/mL) or Gancao extract (1 g/mL) containing glycyrrhizic acid (16.35 mg/mL) and liquiritin (22.38 mg/mL). Subsequently, the single extract or mixed extract was dissolved in HBSS (pH = 7.4) to obtain a series of working standard solutions, and the final concentrations of paeoniflorin, glycyrrhizic acid, or liquiritin in the transport samples were 50 µM, 22 µM, or 54 µM, respectively. In addition, the ratio of Shaoyao and Gancao in the mixed extract was 1:1. Meanwhile, testosterone (internal standard) was dissolved in acetonitrile with acetic acid (v/v = 94/6) to 100 µM. In addition, verapamil was dissolved in water to 5 mM when it was used.

### 3.4. Transport Experiments across the Caco-2 Cell Culture Model

The cell monolayers were washed three times with 37 °C HBSS (pH 7.4). The monolayers were incubated with the buffer for 1 h, and the incubation medium was then aspirated. Afterwards, a single component or extract solution was loaded on the apical or basolateral side. When the transport inhibitor (verapamil) was used, it was loaded only at the donor side. The amounts of transported drugs in the receiver media were determined by ultra-performance liquid chromatography (UPLC). In the test for each solution, four donor samples (400 μL) and four receiver samples (400 μL) were taken at time intervals of 1, 2, 3, and 4 h, followed by an immediate replacement with fresh donor solution (400 μL) to the donor side or fresh buffer (400 μL) to the receiver side. 

To each transport sample (400 μL), 100 μL of acetonitrile containing 100 μM of testosterone was added as an internal standard and a preservative. The resulting mixture was vortexed for 30 s and then centrifuged at 15,000 rpm for 15 min, and the supernatant obtained was analyzed by UPLC.

### 3.5. UPLC Analysis of Transport Samples

**Figure 2:**
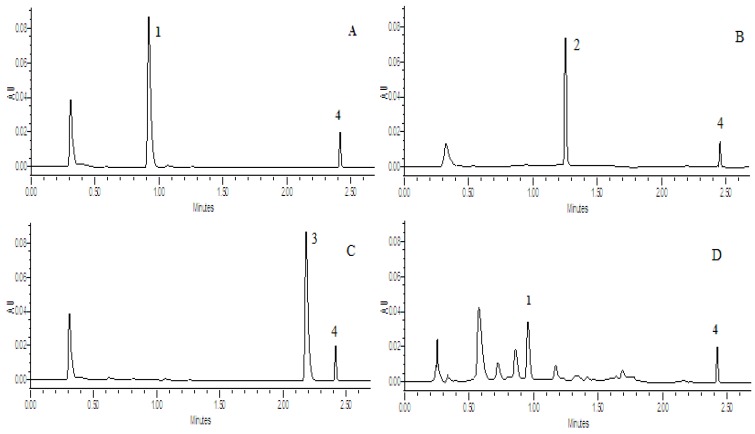

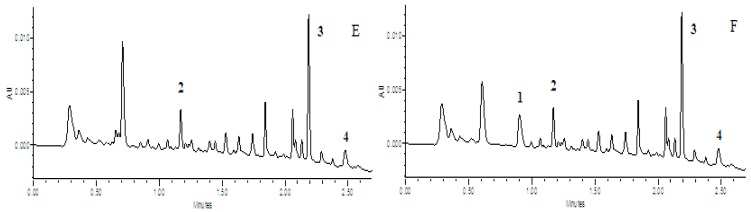
UPLC chromatograms of compounds transported across Caco-2 cell monolayer. (**A**): Paeoniflorin; (**B**): Glycyrrhizic acid; (**C**): Liquiritin; (**D**): Shaoyao extract; (**E**): Gancao extract; (**F**): mixture of Shaoyao and Gancao extract. **1**: paeoniflorin; **2**: glycyrrhizic acid; **3**: liquiritin; **4**: testosterone.

UPLC was used to determine the concentration of the compounds in the transport samples. The conditions for analysis were as follows: system, Waters Acquity UPLC with photodiode array detector and Empower software; column, Acquity UPLC BEH C18, 1.7 μm, 2.1 × 50 mm (Waters, Milford, MA, USA); mobile phase A, acetonitrile; mobile phase B, 0.045% (v/v) phosphoric acid plus 0.06% (v/v) triethylamine in water; gradient, 0 to 0.8 min, 15% A, 0.8 to 1.5 min,15% to 22% A, 1.5 to 1.8 min, 22% to 45% A, 1.8 to 2.2 min, 45% to 60% A, 2.2 to 2.6 min, 60% to 80% A; flow rate, 0.4 mL/min; wavelength, paeoniflorin (231 nm), glycyrrhizic acid (250 nm), liquiritin (276 nm), internal standard (245 nm); injection volume, 5 μL. The retention times for paeoniflorin, liquiritin, glycyrrhizic acid and internal standard were 0.95, 1.25, 2.20 and 2.43 min, respectively. In general, this method was selective and reproducible with day to day variability less than 2%. The tested linear response ranges for all compouds were 0.3125 to 120 μM. The accuracy and precision were greater than 98%. The recovery of three different concentrations was around 97.25%–99.08%, and RSD was less than 5% (n = 5). The UPLC chromatograms were seen in Figure 2. 

### 3.6. Data Analysis

In the Caco-2 cell model, rate of transport is obtained from amount transported *versus* time curve using linear regression. The permeability of a compound is calculated using the following equation:

(1) $$p_{app}=\frac{dQ}{dt\cdot C_{o}\cdot A}$$

where dQ/dt is the rate of drug transport (μM/s), A is the surface area of the cell monolayer (4.2 cm^2^) and C_0_ is the initial concentration in the donor solution (μM/L). All data were presented as means ± SD. Statistical comparisons were evaluated by ANOVA test using the SPSS 16 software. Results were considered significant at *p* < 0.05.

## 4. Conclusions

In the present study, we investigated the absorption and interaction of the main components in the Shaoyao-Gancao drug pair. The absorption of single component compounds (paeoniflorin, glycyrrhizic acid, and liquiritin), the corresponding compounds in the single extract, and those in the extract mixture were compared for the first time using the Caco-2 cell model. On the basis of our results, we can conclude that the absorption of single components paeoniflorin, glycyrrhizic acid, and liquiritin was poor, which may be responsible for their poor intrinsic permeation and transporter-mediated efflux. However, when administrated as an extract, the absorption of paeoniflorin and liquiritin was improved; in particular, concomitant administration of Shaoyao and Gancao extract showed a significant increase in the absorption of paeoniflorin and glycyrrhizic acid. For TCMs, extracts or mixtures but not single components are often used clinically. Our findings clearly showed that extracts or mixtures might promote the absorption of the bioactive compounds in the intestine and thus enhance the efficacy of the TCMs, which reveals the mechanism of TCM compatibility to some extent.

## Figures and Tables

**Table 1:** Permeability of paeoniflorin, glycyrrhizic acid, and liquiritin.

Compound	Concentration /μM	P_app_(×10^−6^ cm/s)		Efflux ratio
		P_AB_	P_BA_	P_BA_/P_AB_
Paeoniflorin	50	0.61 ± 0.09	1.28 ± 0.05 *	2.10
Glycyrrhizic acid	20	0.36 ± 0.04	0.83 ± 0.07 *	2.31
Liquiritin	50	0.73 ± 0.04	1.57 ± 0.05 *	2.15

Data are expressed as mean ± SD (n = 3), * *p* < 0.05, P_AB_ *vs.* P_BA_.

**Table 2:** Permeability of paeoniflorin in Shaoyao extract and glycyrrhizic acid, liquiritin in Gancao extract.

Compound	Concentration /μM	*P_app_*(×10^−6^ cm/s)		Efflux ratio
		P_AB_	P_BA_	P_BA_/P_AB_
Paeoniflorin	50	0.94 ± 0.11 *	1.30 ± 0.02	1.08
Glycyrrhizic acid	22	0.41 ± 0.05	0.89 ± 0.04	2.17
Liquiritin	54	1.20 ± 0.06 *	1.59 ± 0.02	1.33

Data are expressed as mean ± SD (n = 3), * *p* < 0.05, extract *vs.* single component.

**Table 3:** Permeability of paeoniflorin, glycyrrhizic acid, liquiritin in the mixture of Shaoyao-Gancao extract.

Compound	Concentration /μM	*P_app_*(×10^−6^ cm/s)		Efflux ratio
		P_AB_	P_BA_	P_BA_/P_AB_
Paeoniflorin	50	1.96 ± 0.08 **	1.77 ± 0.07 *	0.90
Glycyrrhizic acid	22	1.17 ± 0.08 **	1.21 ± 0.06 *	1.03
Liquiritin	54	1.31 ± 0.06	1.58 ± 0.04	1.20

Data are expressed as mean ± SD (n = 3), * *p* < 0.05, ** *p* < 0.01, component in mixture *vs.* component in single extract.
